# Author’s Response

**DOI:** 10.1308/003588414X13824511649490

**Published:** 2014-01

**Authors:** V Khanduja

**Affiliations:** Cambridge University Hospitals NHS Foundation Trust,UK

Many thanks for your interest in our article. We agree on the issue of multimodal surveys and, certainly, for non-responders, a multimodal survey is a good tool. The other points mentioned in your letter have already been covered in our article. Finally, we do not agree on the issue of surveys being time consuming but not expensive. Somebody is paying for the time!

